# Emergence of knock-down resistance in the *Anopheles gambiae* complex in the Upper River Region, The Gambia, and its relationship with malaria infection in children

**DOI:** 10.1186/s12936-018-2348-8

**Published:** 2018-05-18

**Authors:** Anne L. Wilson, Margaret Pinder, John Bradley, Martin J. Donnelly, Majidah Hamid-Adiamoh, Lamin B. S. Jarju, Musa Jawara, David Jeffries, Ballah Kandeh, Emily J. Rippon, Kolawole Salami, Umberto D’Alessandro, Steven W. Lindsay

**Affiliations:** 1 Durham University, Durham, UK; 2 London School of Hygiene and Tropical Medicine, London, UK; 3 Liverpool School of Tropical Medicine, Liverpool, UK; 4 Medical Research Council Unit The Gambia at the London School of Hygiene & Tropical Medicine, Banjul, The Gambia; 5 National Malaria Control Programme, Banjul, The Gambia

**Keywords:** *Anopheles gambiae*, Insecticide Resistance, Target site resistance, Knockdown resistance, Gambia, Malaria

## Abstract

**Background:**

Insecticide resistance threatens malaria control in sub-Saharan Africa. Knockdown resistance to pyrethroids and organochlorines in *Anopheles gambiae* sensu lato (*s.l.)* is commonly caused by mutations in the gene encoding a voltage-gated sodium channel which is the target site for the insecticide. The study aimed to examine risk factors for knockdown resistance in *An. gambiae s.l.* and its relationship with malaria infection in children in rural Gambia. Point mutations at the *Vgsc*-*1014* locus, were measured in *An. gambiae s.l.* during a 2-year trial. Cross-sectional surveys were conducted at the end of the transmission season to measure malaria infection in children aged 6 months–14 years.

**Results:**

Whilst few *Anopheles arabiensis* and *Anopheles coluzzii* had *Vgsc*-*1014* mutations, the proportion of *An. gambiae* sensu stricto *(s.s.)* mosquitoes homozygous for the *Vgsc*-*1014F* mutation increased from 64.8 to 90.9% during the study. The *Vgsc*-*1014S* or *1014F* mutation was 80% higher in 2011 compared to 2010, and 27% higher in the villages with indoor residual spraying compared to those without. An increase in the proportion of *An. gambiae s.l.* mosquitoes with homozygous *Vgsc*-*1014F* mutations and an increase in the proportion of *An. gambiae s.s.* in a cluster were each associated with increased childhood malaria infection. Homozygous *Vgsc*-*1014F* mutations were, however, most common in *An. gambiae s.s.* and almost reached saturation during the study meaning that the two variables were colinear.

**Conclusions:**

As a result of colinearity between homozygous *Vgsc*-*1014F* mutations and *An. gambiae s.s*., it was not possible to determine whether insecticide resistance or species composition increased the risk of childhood malaria infection.

**Electronic supplementary material:**

The online version of this article (10.1186/s12936-018-2348-8) contains supplementary material, which is available to authorized users.

## Background

Between 2000 and 2015, the prevalence of *Plasmodium falciparum* infection in sub-Saharan Africa (SSA) has halved due to the mass deployment of long-lasting insecticidal nets (LLINs), and to a lesser extent, indoor residual spraying (IRS) [1]. This has, however, increased selection pressure for insecticide resistance in malaria vectors, particularly resistance to pyrethroids, the only insecticide class used currently for treating bed nets. The strength and distribution of insecticide resistance has increased over time and there is growing concern that this will lead to control failure, which has the potential to reverse many of the gains seen in malaria control [2]. One of several mechanisms through which mosquitoes become resistant to insecticides is through mutations in the insecticide target site. Three different point mutations in the voltage-gated sodium channel gene confer knockdown resistance (*kdr*) to pyrethroids and organochlorines such as dichlorodiphenyltrichloroethane (DDT) in *Anopheles gambiae s.l.* [3–5].

There has been little longitudinal insecticide resistance monitoring in The Gambia, but the general impression is that levels of insecticide resistance are low, but rising. Shortly after the introduction of permethrin-treated bed nets in The Gambia in the early 1990s, there was little or no resistance to DDT or permethrin [6, 7]. Prior to the nationwide DDT IRS campaign in 2009, DDT resistance was found in one site bordering Senegal, but mosquitoes from the site in the Upper River Region (URR) were fully susceptible to permethrin, deltamethrin and DDT [8]. Tests performed during a cluster-randomised controlled trial in the URR which compared the efficacy of LLINs versus LLINs and IRS with DDT against malaria in children found complete susceptibility of *An. gambiae s.l.* to DDT and permethrin in 2010 and some loss of susceptibility the following year [9]. Another study, conducted in the same year but outside the study area indicates that there may be pockets of high resistance in the URR [10]. Research also suggests that insecticide resistance may be partly responsible for the heterogeneities in malaria transmission across the country [11]. Although malaria has been declining in The Gambia since 2000 [12, 13], there is continued moderately high seasonal transmission in the URR despite high vector control coverage [14].

The study aimed to examine risk factors for *kdr* resistance in *An. gambiae s.l.* and its relationship with malaria infection in children in The Gambia.

## Methods

### Study site

The study was conducted in the URR (regional capital: Basse Santa Su, 13.3167°N, − 14.2167°W), a rural area of open Sudanian savannah which is divided into north and south banks by the River Gambia. Malaria transmission is highly seasonal being associated with the annual rains which occur from June to October. LLIN use by study children was 55% at baseline and IRS with DDT was implemented in 2009, the year prior to study start.

### Data collection

This secondary analysis uses data from a cluster-randomised controlled trial assessing the efficacy of LLINs versus LLINs and IRS with DDT against malaria among children aged 6 months–14 years in the URR of The Gambia [9]. The study design and results are described in full elsewhere [9, 15]. In brief, 70 clusters of villages were randomly allocated to receive either LLINs or LLINs plus IRS. Permethrin-treated LLINs (2%; Olyset Nets, Sumitomo Chemicals, Japan) were distributed in both arms at the start of the 2010 transmission season to achieve high coverage of sleeping places. IRS with DDT (2 g/m^2^, DDT 75% wettable powder; Hindustan Insecticides, New Delhi, India) was applied to dwelling rooms at the start of each transmission season in the IRS-LLIN arm. Surveys conducted at the end of the transmission season in 2010 and 2011 measured the prevalence of *P. falciparum* infection in a cohort of children.

**Fig. 1:**
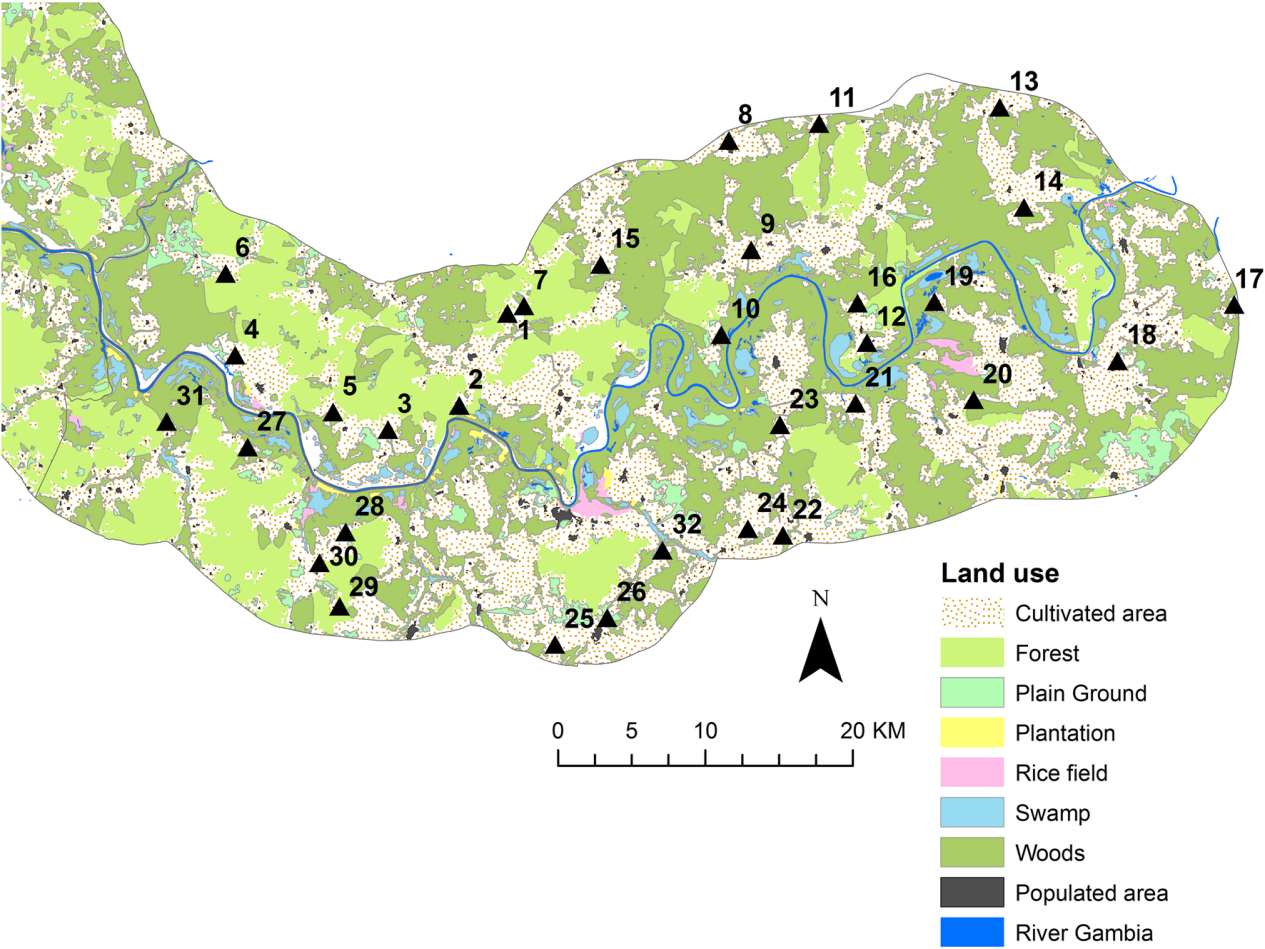
Spatial distribution of 32 entomological sampling sites in the Upper River Region of The Gambia, in relation to landcover/use

Entomological data were collected in 32 clusters (16 in each arm) in six sentinel rooms per cluster. Clusters were chosen purposively for logistical reasons (Fig. 1). Sampling was performed monthly from June to the end of December in 2010 and 2011, and every 2 months during the intervening dry season. Mosquitoes were collected overnight from sentinel rooms in which an adult slept under an LLIN using a CDC light trap. The epidemiological dataset was restricted to children who resided in the entomological clusters (1543 children in 2010 and 1564 children in 2011).

Mosquitoes were identified to species using established keys [16, 17]. Sub-species (*Anopheles arabiensis*, *Anopheles coluzzii*, *An. gambiae s.s.* and hybrid *An. gambiae s.s.* x *An. coluzzii* form, hereafter termed ‘hybrid’) and genotype at the *Vgsc*-*1014* locus were determined according to previously described molecular methods [18–20]. Leucine to serine (*Vgsc*-*1014S*, previously termed *kdr*-east) or leucine to phenylalanine (*Vgsc*-*1014F*, previously termed *kdr*-west) mutations at this locus confer *kdr*.

### Mapping and spatial analysis

Digitised maps produced by the Japan International Cooperation Agency under The Japanese Government Technical Cooperation Programme and The Government of the Republic of The Gambia from 2002 were obtained. Global Moran’s spatial autocorrelation coefficient, I, was calculated at 1 km intervals between 9 km (the shortest distance at which all sampling point locations had at least one neighbour) and 25 km to examine spatial independence in species distributions. The z-score returned indicated the intensity of clustering. Mapping and spatial analysis was performed using ArcGIS^®^ software (Release 10.4.1, Environmental Systems Research Institute: Redlands, CA).

### Statistical analysis

Transmission seasons were defined as 16 August–31 December 2010 and 15 August 2011–1 January 2012 to avoid the months prior to and during application of IRS and the intervening dry season. Proportions of mosquitoes by species and *kdr* status over time and by village were calculated. Mixed effect logistic regression models including cluster as a random effect were used to determine the relationship between vector species of individual mosquitoes and Euclidean distance of the cluster from the River Gambia, and secondly, the effect of variables such as year and study arm on *kdr* status of individual mosquitoes, whilst controlling for species. Variables were tested for departure from linear trend where necessary. Stepwise selection procedures and likelihood ratio tests were used to determine the combination of covariates, which fitted the data best. Mixed effect logistic regression models were also used to look at the effect of i) cluster-level *kdr* status (prevalence of any *Vgsc*-*1014* mutation i.e. any mutation at the *Vgsc*-*1014* locus, and homozygous *Vgsc*-*1014F* mutation by cluster) and ii) prevalence of *An. gambiae* s.s. by cluster on the odds of *P. falciparum* infection in individual children at the end of transmission season surveys, adjusting for clustering and confounding variables. These three explanatory variables were fit as linear variables and expressed as the odds ratio for the effect of a 1 and 10% increase in these variables on the prevalence of malaria infection. R^2^ and the variance inflation factor (VIF) were calculated to identify colinearity between variables. Goodness of fit of models evaluating the effect of cluster level prevalence of either *An. gambiae s.s.* or homozygous *Vgsc*-*1014F* mutations on malaria infection were compared using the Akaike information criterion (AIC). Models were also run to evaluate the effect of absolute numbers of mosquitoes per cluster with any *Vgsc*-*1014* mutation and the homozygous *Vgsc*-*1014F* mutation, and absolute number of *An. gambiae s.s.* on the odds of *P. falciparum* infection in individual children. Statistical analyses were performed using Stata 14 (College Station, TX, USA).

## Results

**Fig. 2:**
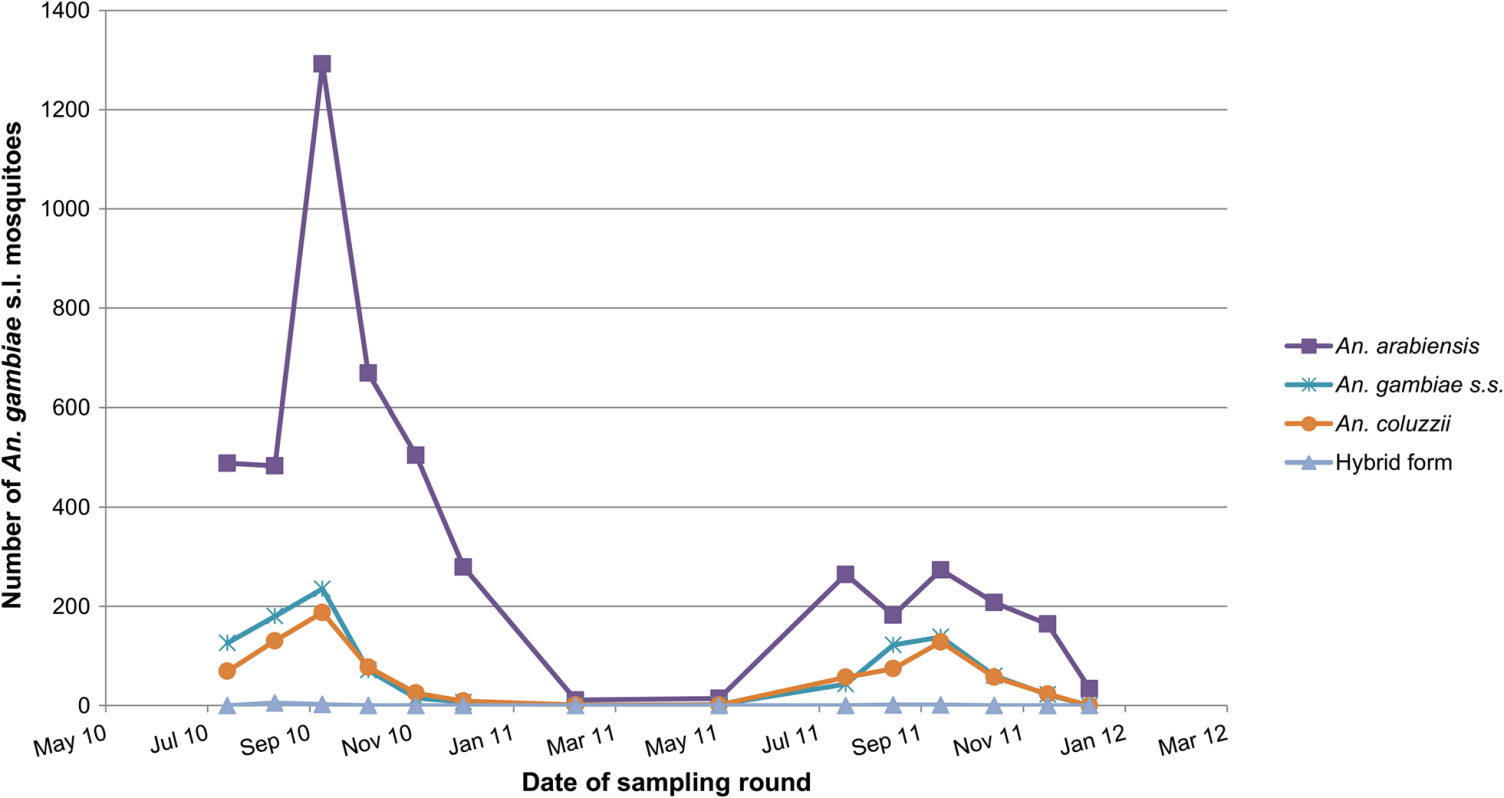
Number of *An. arabiensis*, *An. gambiae s.s., An. coluzzii* and hybrid (*An. gambiae s.s*. × *An. coluzzii*) caught using CDC light traps per round during 2010 and 2011 (IRS using DDT was administered between 15–28 July 2010 and 20 July–9 August 2011)

A total of 6853 *An. gambiae s.l.* were caught in the 32 sampling sites over the two transmission seasons. Of these, 6828 (99.6%) were identified to species: 71.3% were *An. arabiensis*, 15.0% *An. gambiae s.s*., 12.3% *An. coluzzii*, and 0.1% hybrid (Fig. 2). Higher numbers were caught during 2010 when there was unusually high rainfall and extensive flooding, compared to 2011 when flooding was limited to areas beside the river. During the 2010 transmission season, 76.1% of *An. gambiae s.l.* were *An. arabiensis*, 12.0% *An. gambiae s.s*., 10.1% *An. coluzzii* and 0.2% hybrid (Additional file 1). During the 2011 transmission season, 57.8% of *An. gambiae s.l.* caught were *An. arabiensis*, 23.0% *An. gambiae s.s*., 19.0% *An. coluzzii* and 0.1% hybrid. Twenty-nine mosquitoes were caught during the dry season (of these 25 were *An. arabiensis*, 2 *An. gambiae s.s*. and 2 *An. coluzzii*). Spatial autocorrelation was found in species distributions with peak autocorrelation operating between 9 and 14 km depending on the species and year.

**Fig. 3:**
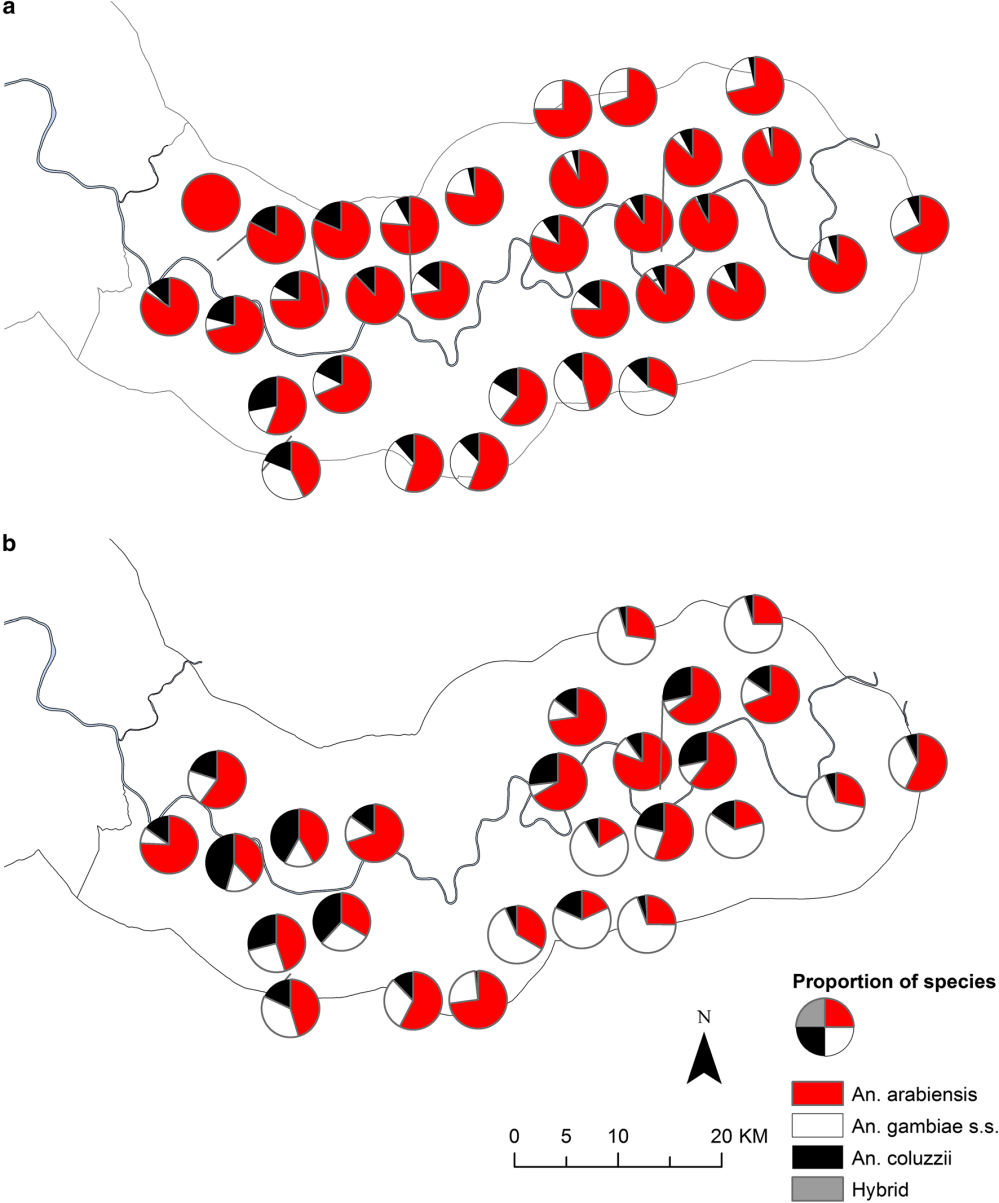
Distribution of members of the *An. gambiae s.l.* complex in the study area during 2010 (**a**) and 2011 (**b**) transmission seasons. Pie charts show percentage composition of species of *An. gambiae s.l.* complex at CDC light trap sampling sites, (excluding sampling sites with less than 10 mosquitoes caught in total across each transmission season)

**Fig. 4:**
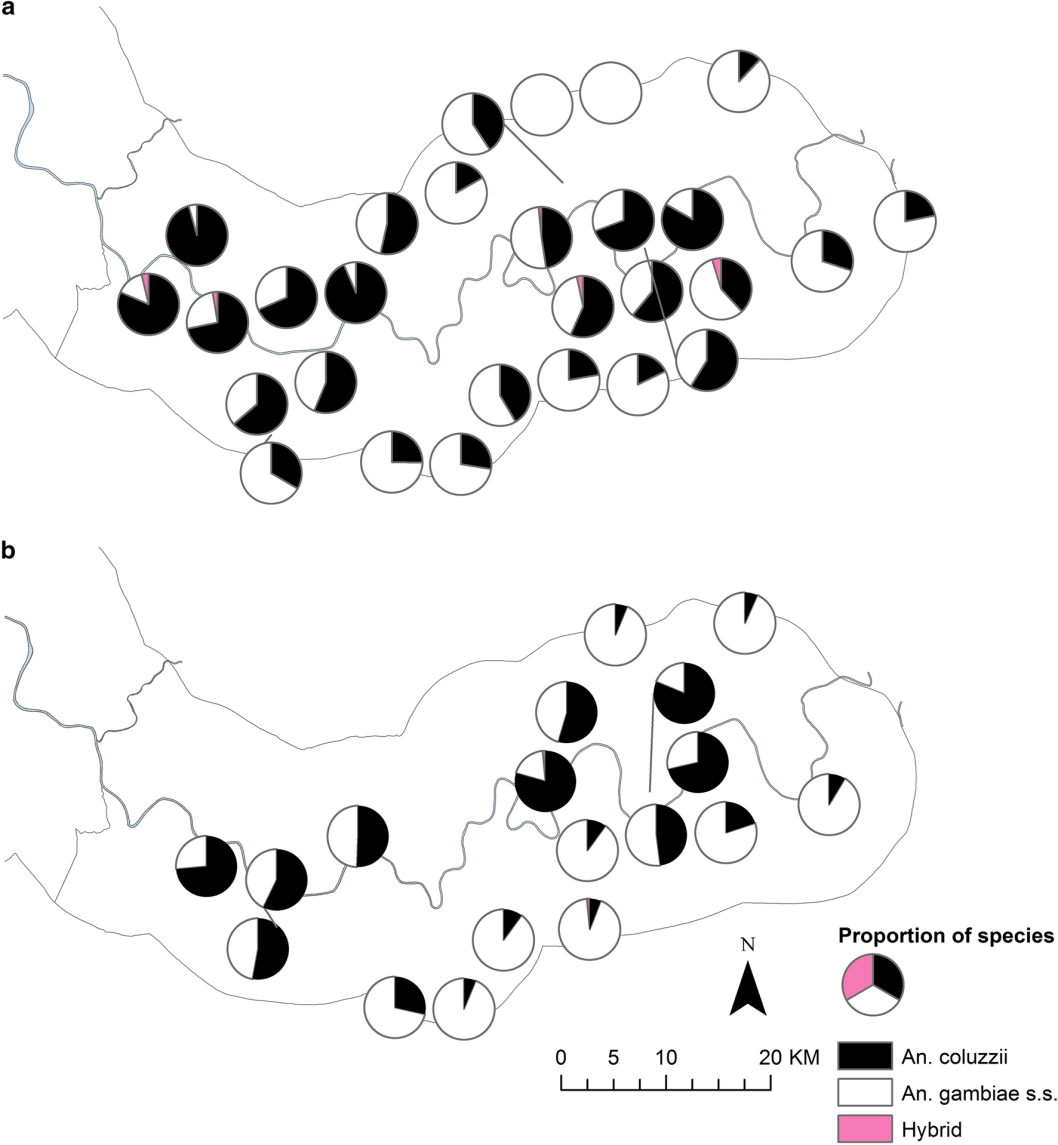
Distribution of members of the *An. gambiae s.l.* species complex (excluding *An. arabiensis*) in the study area during 2010 (**a**) and 2011 (**b**) transmission seasons. Pie charts show percentage *An. gambiae s.l.* species composition (excluding *An. arabiensis*) at CDC light trap sampling sites (excluding sampling sites with less than 10 mosquitoes caught in total across each transmission season)

Analysis of species distributions over the two transmission seasons, showed that *An. gambiae s.s.* was more common further away from the river (Odds ratio, OR for every km away from the river = 1.29, 95% CI 1.21–1.38, p < 0.001) (Figs. 3, 4). Conversely, both *An. arabiensis* and *An. coluzzii* were more common closer to the river (*An. arabiensis* OR = 0.88, 95% CI 0.83–0.94, p < 0.001; *An. coluzzii* OR = 0.91, 95% CI 0.85–0.98, p = 0.01). Similar patterns were found when each year was analysed separately.

In 2010, *An. arabiensis* comprised 81.8% of mosquitoes caught in the LLIN only arm (63.3% in 2011) and 67.8% in the IRS-LLIN arm (50.3% in 2011). As a result, there was a significantly lower odds of collecting *An. arabiensis* in the double intervention arm compared to the LLIN arm of the study in both years (2010: odds ratio, OR = 0.51, 95% CI 0.31–0.84, p = 0.008; 2011: OR = 0.44, 95% CI 0.25–0.78, p = 0.005). In 2010, *An. gambiae s.s*. comprised 6.5% of mosquitoes in the LLIN only arm (17.9% in 2011) and 19.8% in the IRS-LLIN arm (29.9% in 2011). There was a significantly higher odds of finding *An. gambiae s.s.* in the IRS-LLIN arm compared to the LLIN arm of the study in both years (2010: OR = 3.02, 95% CI 1.38–6.57, p = 0.005; 2011: OR = 2.71, 95% CI 1.18–6.19, p = 0.02). There was no difference in the odds of catching *An. coluzzii* between the two study arms in both 2010 and 2011 (2010: OR = 1.12, 95% CI 0.71–1.79, p = 0.63; 2011: OR = 0.81, 95% CI 0.41–1.60, p = 0.54), nor was there a difference in the odds of catching hybrids between the two study arms in 2010 (OR = 1.93, 95% CI 0.43–8.62, p = 0.39).

*Vgsc*-*1014* mutations were found in all species sampled but at differing levels*. An. arabiensis* were predominantly wild-type (73.1% during 2010 and 58.1% during 2011), although the proportion with heterozygous *Vgsc*-*1014S* mutations increased from 20.5% in 2010 to 28.3% in 2011 (OR = 1.58, 95% CI 1.32–1.89, p < 0.001) (Table 1). *Anopheles gambiae s.s.* were predominantly homozygous *Vgsc*-*1014F* and this proportion increased almost to saturation from 64.8% in 2010 to 90.9% in 2011 (OR = 8.24, 95% CI 4.99–13.63, p < 0.001). *An. coluzzii* were predominantly wild-type (73.1% during 2010 and 70.9% during 2011).

**Table 1:** Allele frequency of *Vgsc*-*1014* mutations by species in the study area in 2010 and 2011

Species	Year	Allele frequency of *Vgsc*-*1014* mutations										
		Wild type		Heterozygous *Vgsc*-*1014F*		Homozygous *Vgsc*-*1014F*		Heterozygous *Vgsc*-*1014S*		Homozygous *Vgsc*-*1014S*		Total^a^
		n	%	n	%	n	%	n	%	n	%	
*An. arabiensis*	2010	2360	73.1	31	1.0	44	1.4	662	20.5	77	2.4	3227
	2011	500	58.1	18	2.1	23	2.7	243	28.3	39	4.5	860
*An. gambiae* * s.s.*	2010	74	14.6	93	18.3	329	64.8	3	0.6	3	0.6	508
	2011	10	2.9	12	3.5	311	90.9	0	0.0	0	0.0	342
*An. coluzzii*	2010	313	73.1	38	8.9	66	15.4	6	1.4	1	0.2	428
	2011	200	70.9	35	12.4	30	10.6	1	0.4	1	0.4	282
*An. gambiae *x *An. coluzzii *hybrid	2010	1	14.3	3	42.9	3	42.9	0	0	0	0	7
	2011	1	50.0	0	0	1	50.0	0	0	0	0	2

^a^ Including unclassified mosquitoes

In both years, the odds of having any type of *Vgsc*-*1014* mutation was significantly higher in the IRS-LLIN arm compared to the LLIN only arm (2010: OR = 1.54, 95% CI 1.07–2.22, p = 0.02; 2011: OR = 2.26, 95% CI 1.24–4.11, p = 0.01) (Table 2). This was primarily due to the higher proportion of *Vgsc*-*1014F* mutations, particularly homozygous *Vgsc*-*1014F* mutations, in the double intervention compared to the single intervention arm. In 2010, IRS-LLIN villages had 2.24 times the odds of mosquitoes carrying homozygous *Vgsc*-*1014F* mutations compared to LLIN only villages (95% CI 1.12–4.49, p = 0.02), while in 2011, double intervention villages had 2.52 times the odds (95% CI 1.20–5.29, p = 0.01). There was an increased odds of a mosquito carrying the heterozygous *Vgsc*-*1014F* mutation in 2010 (OR = 2.17, 95% CI 1.28–3.68, p = 0.004), but not in 2011 (OR = 1.32, 95% CI 0.75–2.33, p = 0.34). No significant difference in the odds of heterozygous or homozygous *Vgsc*-*1014S* mutations was found between IRS-LLIN villages and LLIN villages in 2010 or 2011.

**Table 2:** Odds ratios of *Vgsc*-*1014* mutations and their association with study arm in 2010 and 2011

*Kdr* mutation status	2010				2011			
	LLIN only arm	IRS-LLIN arm	OR (95% CI) (adjusted for clustering)	p value	LLIN only arm	IRS-LLIN arm	OR (95% CI) (adjusted for clustering)	p value
	n (%)	n (%)			n (%)	n (%)		
Wild type	1761 (70.3%)	1015 (58.5%)			461 (53.9%)	250 (39.6%)		
Any *Vgsc*-*1014* mutation	695 (27.7%)	693 (39.9%)	1.54 (1.07–2.22)	0.02	370 (43.2%)	344 (54.4%)	2.26 (1.24–4.11)	0.01
Heterozygous *Vgsc*-*1014F*	70 (2.8%)	103 (5.9%)	2.17 (1.28–3.68)	0.004	34 (4.0%)	31 (4.9%)	1.32 (0.75–2.33)	0.34
Homozygous *Vgsc*-*1014F*	167 (6.7%)	290 (16.7%)	2.24 (1.12–4.49)	0.02	169 (19.7%)	196 (31.0%)	2.52 (1.20–5.29)	0.01
Heterozygous *Vgsc*-*1014S*	412 (16.4%)	262 (15.1%)	0.86 (0.67–1.11)	0.26	149 (17.4%)	95 (15.0%)	0.71 (0.41–1.23)	0.22
Homozygous *Vgsc*-*1014S*	46 (1.8%)	38 (2.2%)	1.09 (0.60–1.99)	0.78	18 (2.1%)	22 (3.5%)	1.34 (0.53–3.36)	0.54
N	2506	1736			856	632		

**Fig. 5:**
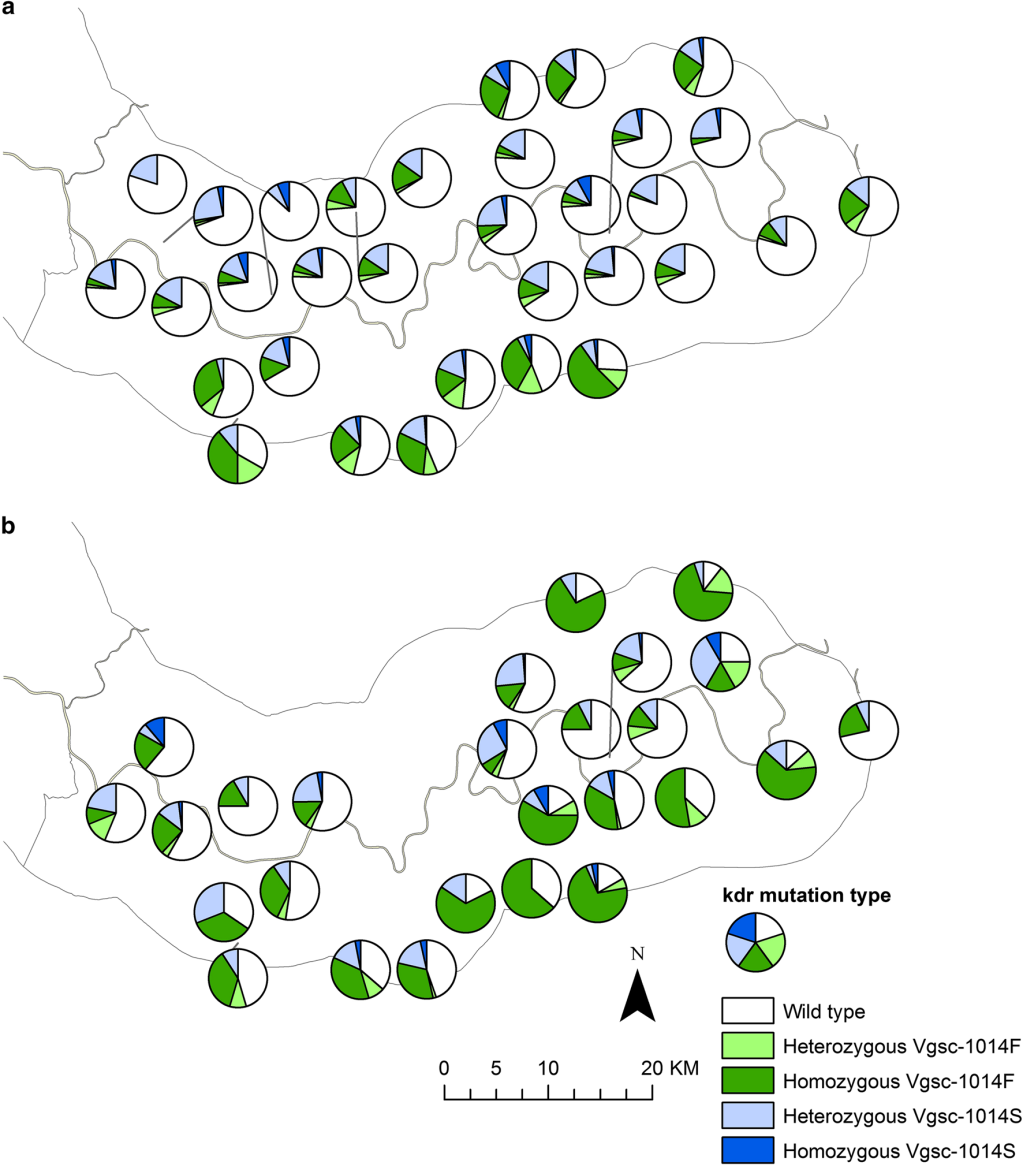
*Vgsc-1014* mutation status of *An. gambiae s.l.* in the study area during 2010 (**a**) and 2011 (**b**) transmission seasons. Pie charts show percentage wildtype, homozygous and heterozygous *Vgsc-1014F* and *Vgsc-1014S* mutations in *An. gambiae s.l.* complex at CDC light trap sampling sites (excluding sampling sites with less than 10 mosquitoes caught in total across each transmission season)

Distribution maps of *Vgsc*-*1014* mutations show an increase in the proportion of mosquitoes carrying homozygous *Vgsc*-*1014F* mutations in villages on the south bank and in the northern part of the study area bordering Senegal between 2010 and 2011 (Fig. 5), which mirrors the increase in the proportion of *An. gambiae s.s.* in these areas (Fig. 3, 4).

In a multivariable model, species, study arm and year of survey were associated with odds of any *Vgsc*-*1014* mutation (Table 3). Adjusting for year and study arm, *An. gambiae s.s*. mosquitoes had 18.49 times the odds of having any *Vgsc*-*1014* mutation compared to *An. arabiensis* (95% CI 14.48–23.61, p < 0.001), while *An. coluzzii* had 0.76 times the odds of having any *Vgsc*-*1014* mutation (95% CI 0.63–0.92, p = 0.004) compared to *An. arabiensis*. Adjusting for species and year, mosquitoes caught in the LLIN-IRS arm had 1.27 times the odds of having any *Vgsc*-*1014* mutation compared to mosquitoes in the LLIN only arm (95% CI 1.03–1.55, p = 0.02). Adjusting for species and study arm, mosquitoes caught in 2011 had 1.80 times the odds of having any *Vgsc*-*1014* mutation compared to those caught in 2010 (95% CI 1.56–2.09, p < 0.001).

**Table 3:** Association between explanatory variables and the odds ratio of having any *Vgsc*-*1014* mutation

Variable	Proportion with any *Vgsc*-*1014* mutation n/N (%)	Univariable analysis (adjusted for clustering on village)			Multivariable analysis		
		OR	95% CI	p value	OR	95% CI	p value
Species							
*An. arabiensis*	1137/3997 (28.4%)	1					
*An. gambiae s.s.*	751/835 (89.9%)	20.31	15.92–25.91	< 0.001	18.49	14.48–23.61	< 0.001
*An. coluzzii*	178/691 (25.8%)	0.84	0.70–1.02	0.08	0.76	0.63–0.92	0.004
Study arm							
LLIN only	1065/3287 (32.4%)	1					
DDT-IRS and LLIN	1037/2302 (45.0%)	1.50	1.04–2.17	0.03	1.27	1.03–1.55	0.02
Year							
2010	1388/4164 (33.3%)	1					
2011	714/1425 (50.1%)	2.08	1.82–2.37	< 0.001	1.80	1.56–2.09	< 0.001
Distance from the river (km)	–	1.13	1.08–1.18	< 0.001			
Bank of River Gambia							
North	975/2863 (34.1%)	1					
South	1127/2726 (41.3%)	1.60	1.10–2.33	0.02			
Parity in village (%)	–	1.19	0.07–21.47	0.91			
Mean female *An. gambiae* s.l. density trap/night/village	–	0.94	0.86–1.03	0.17			
LLIN use in village (%)	–	0.70	0.02–22.22	0.84			

*CI* confidence interval, *OR* odds ratio

*P. falciparum* infection status was ascertained for 1543 children at the end of the 2010 transmission season and 1564 children in 2011. Multivariable analysis showed that girls were more likely to be infected with *P. falciparum* in 2010 (OR = 1.47, 95% CI 1.08–1.98, p = 0.01), but were less likely to be infected in 2011 (OR = 0.73, 95% CI 0.56–0.96, p = 0.02) (Table 4). Older children were more likely to be infected with *P. falciparum* at the end of the transmission season in both years (OR for 1 year increase in age: 2010 = 1.06, 95% CI 1.02–1.10, p = 0.007, 2011 = 1.12, 95% CI 1.08–1.17, p < 0.001). In 2011, children sleeping under an LLIN the previous night were less likely to be infected than children who had not slept under an LLIN (OR = 0.49, 95% CI 0.28–0.83, p = 0.009), although there was no significant association between LLIN use and infection in 2010. There was no significant association between cluster level prevalence of any *Vgsc*-*1014F* mutation and malaria infection in children in either year. In univariable analysis there was a tendency towards an increased odds of *P. falciparum* infection among children living in clusters with a high proportion of mosquitoes specifically carrying homozygous *Vgsc*-*1014F* mutations (OR for a 10% increase in the proportion of *An. gambiae s.l.* mosquitoes with any *Vgsc*-*1014* mutation 2010: OR = 1.25, 95% CI 0.99–1.58, p = 0.07, 2011: OR = 1.13, 95% CI 1.01–1.27, p = 0.04). There was also a similar magnitude increase in the odds of *P. falciparum* infection among children living in clusters with a high proportion of mosquitoes identified as *An. gambiae s.s.* in univariable analysis (OR for a 10% increase in the proportion of *An. gambiae s.l*. mosquitoes identified as *An. gambiae s.s.* 2010: OR = 1.27, 95% CI 1.03–1.55, p = 0.02, 2011: OR = 1.12, 95% CI 1.01–1.25, p = 0.03). The proportion of homozygous *Vgsc*-*1014F* mutations and proportion of *An. gambiae s.s.* at each cluster were colinear (2010: R^2^ = 0.94, VIF = 17.3, 2011: R^2^ = 0.91, VIF = 10.8). As a result, these variables could not be combined in the multivariable model. Comparison of the AIC for models including either cluster level proportion of *An. gambiae s.s.* or mosquitoes carrying homozygous *Vgsc*-*1014F* mutations was not able to distinguish which model provided better goodness of fit (2010: AIC for model including *An. gambiae s.s*. = 1195.38, AIC for model including homozygous *Vgsc*-*1014F* mutations = 1196.90, 2011: AIC for model including *An. gambiae s.s. *= 1413.25, AIC for model including homozygous *Vgsc*-*1014F* mutations = 1413.71). Looking at total number of mosquitoes collected in each cluster, rather than percentage composition, there was no significant association between either the absolute number of *An. gambiae s.s.* mosquitoes, mosquitoes with *Vgsc*-*1014* mutations or mosquitoes with homozygous *Vgsc*-*101F* mutations and malaria infection in children at the end of the transmission season in both years.

**Table 4:** Association between explanatory variables and odds ratio of *P. falciparum* infection in children

Variable	2010 (end of transmission season)					2011 (end of transmission season)				
	*P. falciparum* infection at end of season n/N (%)	Univariable analysis		Multivariable analysis		*P. falciparum* infection at end of season n/N (%)	Univariable analysis		Multivariable analysis	
		OR (95% CI)	p value	OR (95% CI)	p value		OR (95% CI)	p value	OR (95% CI)	p value
Gender										
Male	99/796 (12.4%)	1	–	1	–	173/836 (20.7%)	1	–	1	–
Female	123/747 (16.5%)	1.45 (1.08–1.96)	0.01	1.47 (1.08–1.98)	0.01	123/728 (16.9%)	0.74 (0.57–0.97)	0.03	0.73 (0.56–0.96)	0.02
Age of child (yrs)	–	1.06 (1.02–1.10)	0.007	1.06 (1.02–1.10)	0.007	–	1.13 (1.08–1.17)	< 0.001	1.12 (1.08–1.17)	< 0.001
Child slept under an LLIN the previous night										
No	20/142 (14.1%)	1				26/83 (31.3%)	1	–		
Yes	202/1400 (14.4%)	1.10 (0.64–1.87)	0.74			268/1479 (18.1%)	0.44 (0.26–0.74)	0.002	0.49 (0.28–0.83)	0.009
Study arm										
LLIN only	87/763 (11.4%)	1	–			153/823 (18.6%)	1	–	1	–
LLIN and IRS	135/780 (17.3%)	1.51 (0.83–2.76)	0.18			143/741 (19.3%)	1.20 (0.69–2.09)	0.53		
Bank of River Gambia										
North	118/812 (14.5%)	1	–			110/760 (14.5%)	1	–		
South	104/731 (14.2%)	1.05 (0.57–1.96)	0.87			186/804 (23.1%)	1.77 (1.05–2.97)	0.03		
House construction										
Open eaves	124/848 (14.6%)	1	–			183/872 (21.0%)	1	–		
Closed eaves	79/609 (13.0%)	0.91 (0.63–1.30)	0.59			113/692 (16.3%)	0.81 (0.60–1.09)	0.16		
Cluster level prevalence of [values in square brackets refer to OR for a 10% increase]										
Any *Vgsc*-*1014* mutation	–	1.02 (1.00–1.04) [1.20 (0.96-1.51)]	0.10			–	1.01 (1.00–1.02) [1.10 (0.97–1.25)]	0.13		
Homozygous *Vgsc*-*1014F*	–	1.02 (1.00–1.05) [1.25 (0.99-1.58)]	0.07	^a^		–	1.01 (1.00–1.02) [1.13 (1.01–1.27)]	0.04	^a^	
* An. gambiae* s.s.	–	1.02 (1.00–1.04) [1.27 (1.03–1.55)]	0.02	^a^		–	1.01 (1.00–1.02) [1.12 (1.01–1.25)]	0.03	^a^	

^a^ Variables colinear

## Discussion

These findings illustrate the temporal and spatial pattern of the *An. gambiae* complex and *Vgsc*-*1014* mutations in the URR of The Gambia and their association with malaria infection in children from 2010 to 2011. To the authors knowledge, this is the first study to adopt a landscape approach with intensive entomological sampling to understand factors related to the distribution of *kdr* in malaria vectors.

As in previous work in the URR [21–24], *An. arabiensis* was the most abundant member of the *An. gambiae* complex and persisted longer into the dry season than the other species. The frequency of hybrids was 0.1–0.2%, a slightly lower proportion than that shown by others in The Gambia [24, 25]. *Anopheles gambiae* s*.s.* were more common in villages away from the River Gambia which corresponds with previous studies that reported *An. gambiae s.s.* prefers small rain-dependent larval habitats on free-draining soil covered with open woodland savannah or farmland [26, 27]. *Anopheles arabiensis* was more common near the river suggesting that their aquatic habitats are common in wetlands, such as rain-fed ricefields, adjacent to the river [28]. Indeed, previous studies found *An. arabiensis* in water bodies along the edge of the alluvial soils, particularly in areas of rice cultivation in the Central River Region [29]. *Anopheles coluzzii* was also more common closer to the river which supports research showing this species exploits semi-permanent aquatic habitats that are also frequented by *An. arabiensis* [26, 30, 31]. Although the literature suggests the distribution of species is likely to be due to differential larval habitats, this result may be because *An. gambiae s.s.* has less flexible host choice behaviours than *An. arabiensis* [32–34] and so contributes a larger proportion of the mosquito catch further away from the river.

Interestingly, fewer *An. arabiensis* were caught in villages in the double intervention compared to the single intervention arm, while the opposite pattern was seen with *An. gambiae s.s.* If the *Vgsc*-*1014F* mutation translates into phenotypic resistance, this may have given *An. gambiae s.s.* a competitive advantage over *An. arabiensis* in the double intervention arm. This is a different pattern from that in East Africa where, with the scale-up of interventions, *An. arabiensis* is starting to dominate over *An. gambiae s.s*. since the latter is more endophagic and endophillic and so is thought to be preferentially killed by LLINs [35–37].

Species, year of survey and study arm were associated with odds of any *Vgsc*-*1014* mutation. There was a 1.27 increase in the odds of any *Vgsc*-*1014* mutation in the double intervention compared to the single intervention arm and a 1.80 increase in the odds of any *Vgsc*-*1014* mutation between 2010 and 2011. The odds of any *Vgsc*-*1014* mutation was 18.49 times higher in *An. gambiae s.s.* compared to *An. arabiensis*. Taken together this suggests that (i) LLINs and DDT used together provide more selection pressure than LLINs alone and (ii) there was an increase in selection pressure over the two years most likely due to the second IRS round, and (iii) selection pressure favours *An. gambiae s.s.* since it has a higher frequency of *kdr*. Several studies have shown an increase in the frequency of *kdr* mutations following implementation of vector control interventions [38–41]. High levels of *kdr* in *An. gambiae s.s.* compared to *An. arabiensis* may be explained by a greater propensity for indoor resting and feeding of *An. gambiae s.s.* and, therefore, potential for increased contact with insecticides on walls or LLINs [17, 34, 42]. The study used DDT for IRS and pyrethroid-treated LLINs, and as such is it unsurprising that selection pressure for development of *Vgsc*-*1014* mutations was high in the double intervention arm. The IRS in the study was performed by government teams using the insecticide selected by the National Malaria Control Programme but an alternative insecticide class, such as an organophosphate or carbamate would have been a better option to reduce selection pressure [10]. In fact, based on insecticide resistance monitoring, the control programme recently started to implement rotation of IRS insecticides beginning with bendiocarb in 2015 and 2016 and pirimiphos-methyl in 2017.

Older children had a higher odds of *P. falciparum* infection in both of the end of season surveys. It is unclear why females were at increased risk of infection in the first survey but at lower risk in the second survey and this may be an anomalous result. In 2011, children sleeping under an LLIN had half the odds of being infected compared to children not sleeping under a net, but no significant difference was observed in 2010. This result is probably due to chance because of low numbers of children not using an LLIN in this study. There was no significant association between the cluster level proportion of mosquitoes with any *Vgsc*-*1014F* mutations and malaria infection in children. However, univariable analysis did show an association between *P. falciparum* infection in children and the cluster level proportion of *An. gambiae s.s.* and homozygous *Vgsc*-*1014F* mutations specifically, especially in the second year of the study. However, due to colinearity between *An. gambiae s.s.* and homozygous *Vgsc*-*1014F* mutations these variables could not be combined in the multivariable model. Calculation of the AIC was not able to distinguish between models including these two variables and so it was not possible to say whether high proportions of *An. gambiae s.s*. or homozygous *Vgsc*-*1014F* mutations increased *P. falciparum* infection in children.

This study has several other limitations. Firstly, the analysis used secondary data which meant that the original study was not primarily designed to measure the spatial epidemiology of *kdr* resistance. Secondly, it did not verify the phenotype of the mosquitoes in bioassays or investigate other resistance markers, such as those involved in metabolic resistance. Insecticide resistance is typically driven by complex interactions between multiple alleles and this dataset only looks at a few alleles. Thirdly, spatial autocorrelation was present in species distributions and therefore the assumption that clusters were independent was false. This may have inflated the value of test statistics and increased the chance of a type I error.

Increased malaria infection in the study children may be explained by differences in the species distribution in the villages, specifically possible higher efficiency transmission by *An. gambiae s.s. Anopheles gambiae s.s.* is a more efficient vector than *An. arabiensis*, although it is less clear whether there is a difference in transmission efficiency between *An. gambiae s.s.* and *An. coluzzii* [43–45]. Alternatively, heterogeneity in malaria infection could also be due to the impact of *kdr*. Indeed, previous studies have highlighted a lack of decline in malaria in the URR [14, 46] and a study of paired high and low malaria prevalence villages in The Gambia suggested that heterogeneous transmission may be partly due to insecticide resistance [11]. Opondo et al. showed that DDT mortality for *An. gambiae s.s.* was significantly lower in high prevalence compared to low prevalence villages and that there was a significant association between the *Vgsc*-*1014F* mutation in *An. gambiae s.s*. and resistance to DDT and deltamethrin [11]. This mutation was a strong predictor of insecticide resistance and effectively masked the effect of other mutations in this study such as those associated with metabolic resistance. However, the role of *kdr* is not clear cut [47, 48] and several studies show that pyrethroid LLINs were still able to kill *An. gambiae* despite high *kdr* frequencies [49–52]. Analysis did not show any significant relationship between childhood malaria infection and the absolute number of *An. gambiae s.s.* mosquitoes, mosquitoes with any *Vgsc*-*1014* mutation or mosquitoes with the homozygous *Vgsc*-*1014F* mutation per cluster. This is most likely because of low vector numbers in some of the village clusters.

## Conclusions

In conclusion, the homozygous *Vgsc*-*1014F* mutation occurred predominantly in *An. gambiae s.s.* and increased almost to saturation during the course of the study. It also occurred at higher frequencies where IRS was used in addition to LLINs, probably because the *kdr* mutation confers a selective advantage in the presence of insecticides. There was a 13% increase in the odds of malaria infection in children associated with a 10% increase in the proportion of *An. gambiae s.l.* carrying the *Vgsc*-*1014F* mutation in 2011. Moreover there was a 27% increase in the odds of malaria infection with a 10% increase in the proportion of *An. gambiae s.s.* mosquitoes in 2010 and a 12% increase in 2011. It was, however, impossible to determine whether resistance or species increased the odds of childhood malaria infection since the homozygous *Vgsc*-*1014F* mutation was colinear with *An. gambiae s.s.*

## Additional file

**Additional file 1.** Characteristics of village clusters and proportion species composition during 2010 and 2011 transmission seasons.

## Abbreviations

AIC: akaike information criterion
CDC: Centers for Disease Control
CI: confidence interval
DDT: dichlorodiphenyltrichloroethane
IRS: indoor residual spraying
*kdr*: knockdown resistance
LLIN: long-lasting insecticidal net
OR: odds ratio
SSA: sub-Saharan Africa
URR: Upper River Region
VIF: variance inflation factor
