# Correction to: Emergence of knock-down resistance in the *Anopheles gambiae* complex in the Upper River Region, The Gambia, and its relationship with malaria infection in children

**DOI:** 10.1186/s12936-018-2374-6

**Published:** 2018-06-07

**Authors:** Anne L. Wilson, Margaret Pinder, John Bradley, Martin J. Donnelly, Majidah Hamid‑Adiamoh, Lamin B. S. Jarju, Musa Jawara, David Jeffries, Ballah Kandeh, Emily J. Rippon, Kolawole Salami, Umberto D’Alessandro, Steven W. Lindsay

**Affiliations:** 1 Durham University, Durham, UK; 2 London School of Hygiene and Tropical Medicine, London, UK; 3 Liverpool School of Tropical Medicine, Liverpool, UK; 4 Medical Research Council Unit The Gambia at the London School of Hygiene & Tropical Medicine, Banjul, The Gambia; 5 National Malaria Control Programme, Banjul, The Gambia

## Correction to: Malar J (2018) 17:205 10.1186/s12936-018-2348-8

Unfortunately, the original article [1] contained an error mistakenly carried forward by the Production department handling this article whereby some figures and their captions were interchanged. The correct figures (Figs. 1, 2, 3, 4, 5) and captions are presented in this erratum. The original article has also been updated to reflect this correction.

**Fig. 1:**
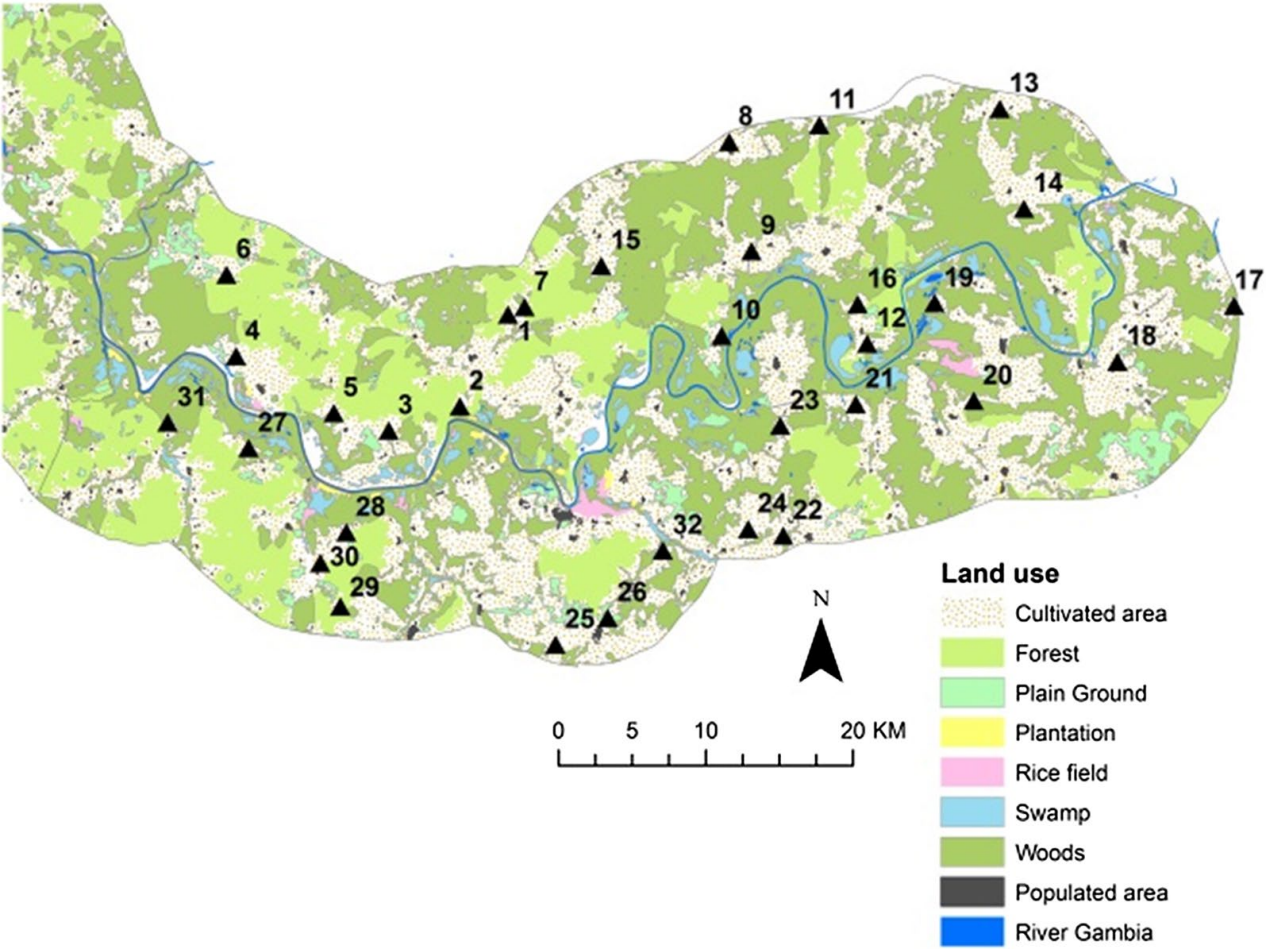
Spatial distribution of 32 entomological sampling sites in the Upper River Region of The Gambia, in relation to landcover/use

**Fig. 2:**
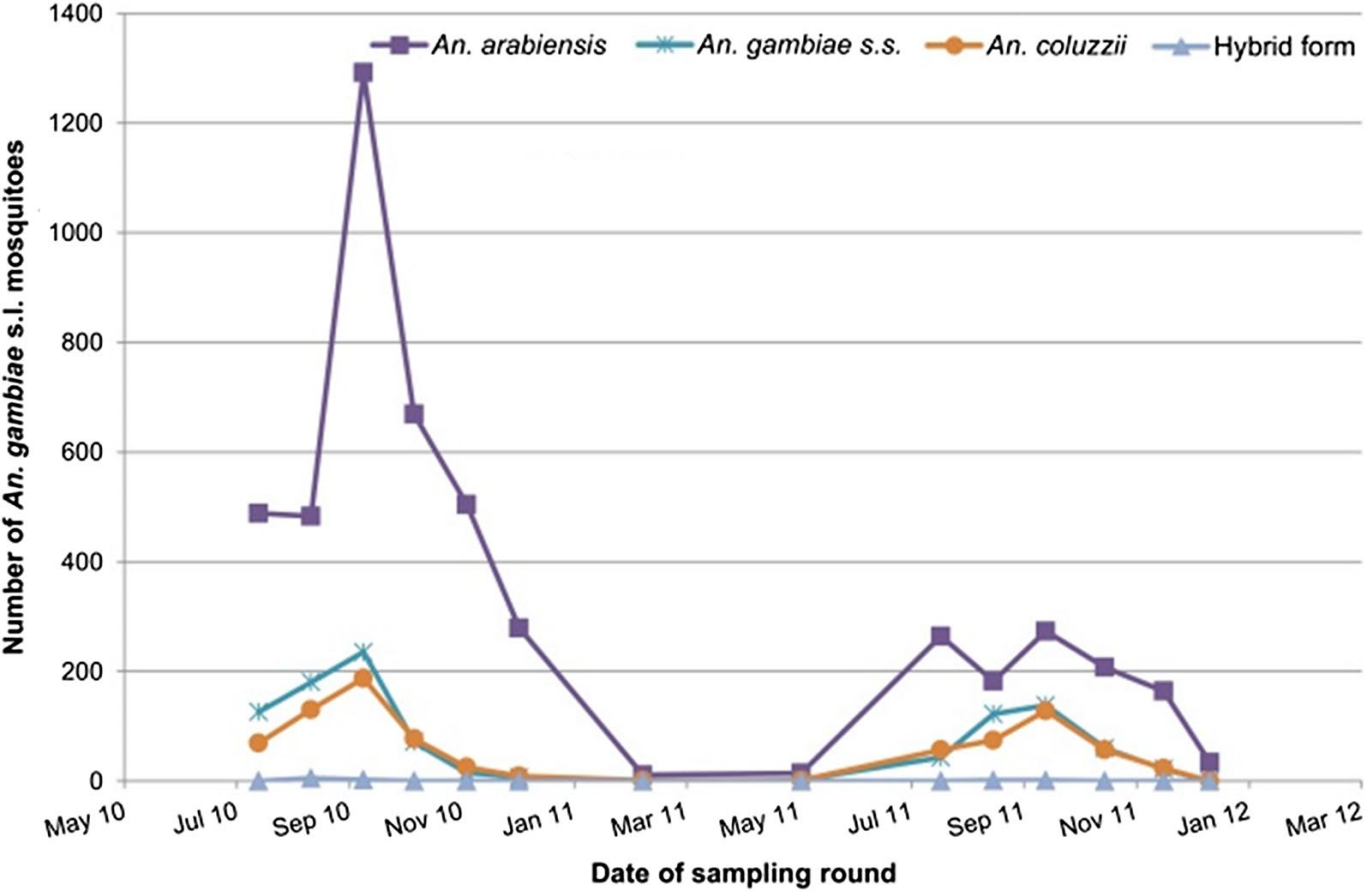
Number of *An. arabiensis*, *An. gambiae s.s., An. coluzzii* and hybrid (*An. gambiae s.s*. × *An. coluzzii*) caught using CDC light traps per round during 2010 and 2011 (IRS using DDT was administered between 15–28 July 2010 and 20 July–9 August 2011)

**Fig. 3:**
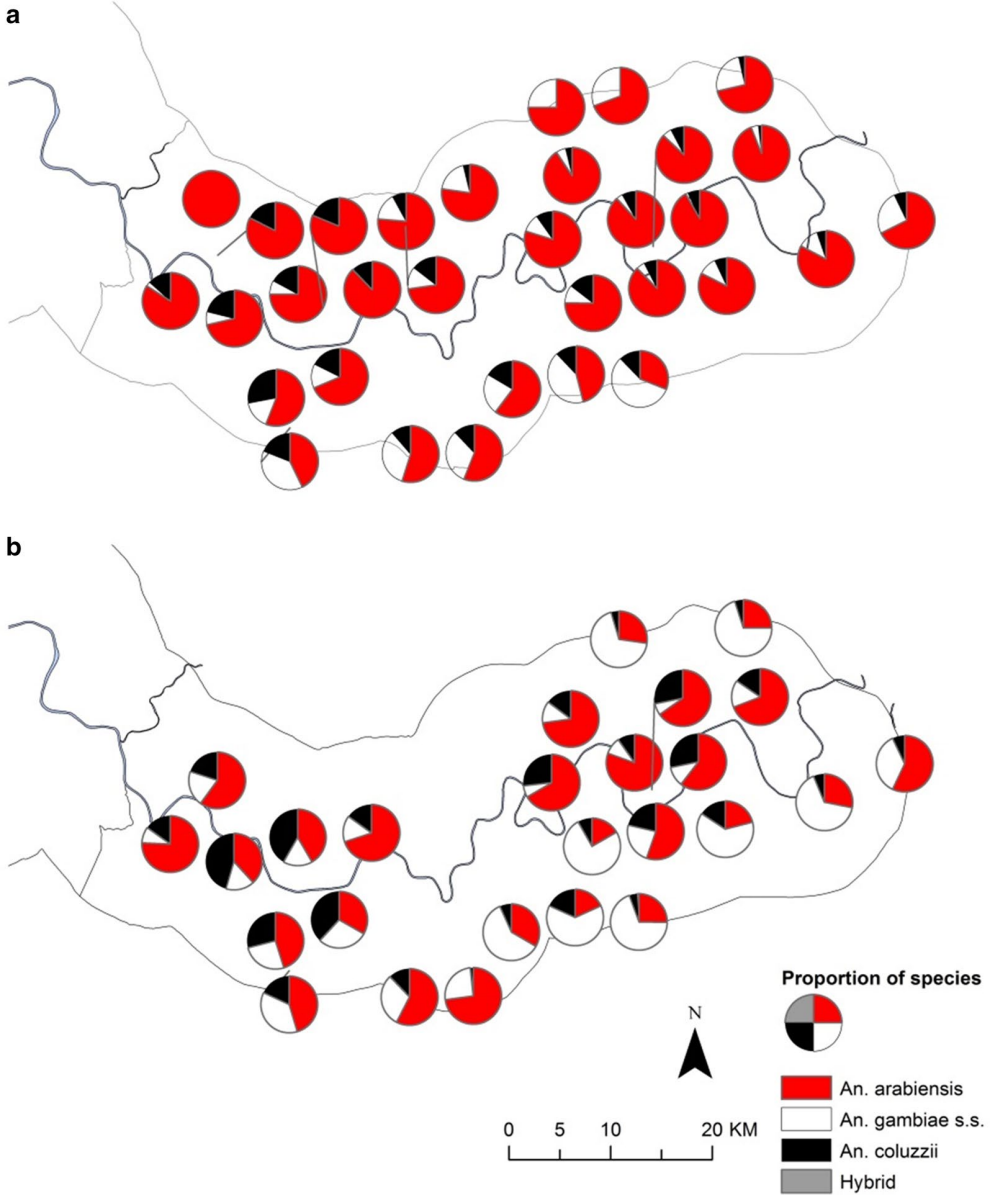
Distribution of members of the *An. gambiae s.l.* complex in the study area during 2010 (**a**) and 2011 (**b**) transmission seasons. Pie charts show percentage composition of species of *An. gambiae s.l.* complex at CDC light trap sampling sites (excluding sampling sites with less than 10 mosquitoes caught in total across each transmission season)

**Fig. 4:**
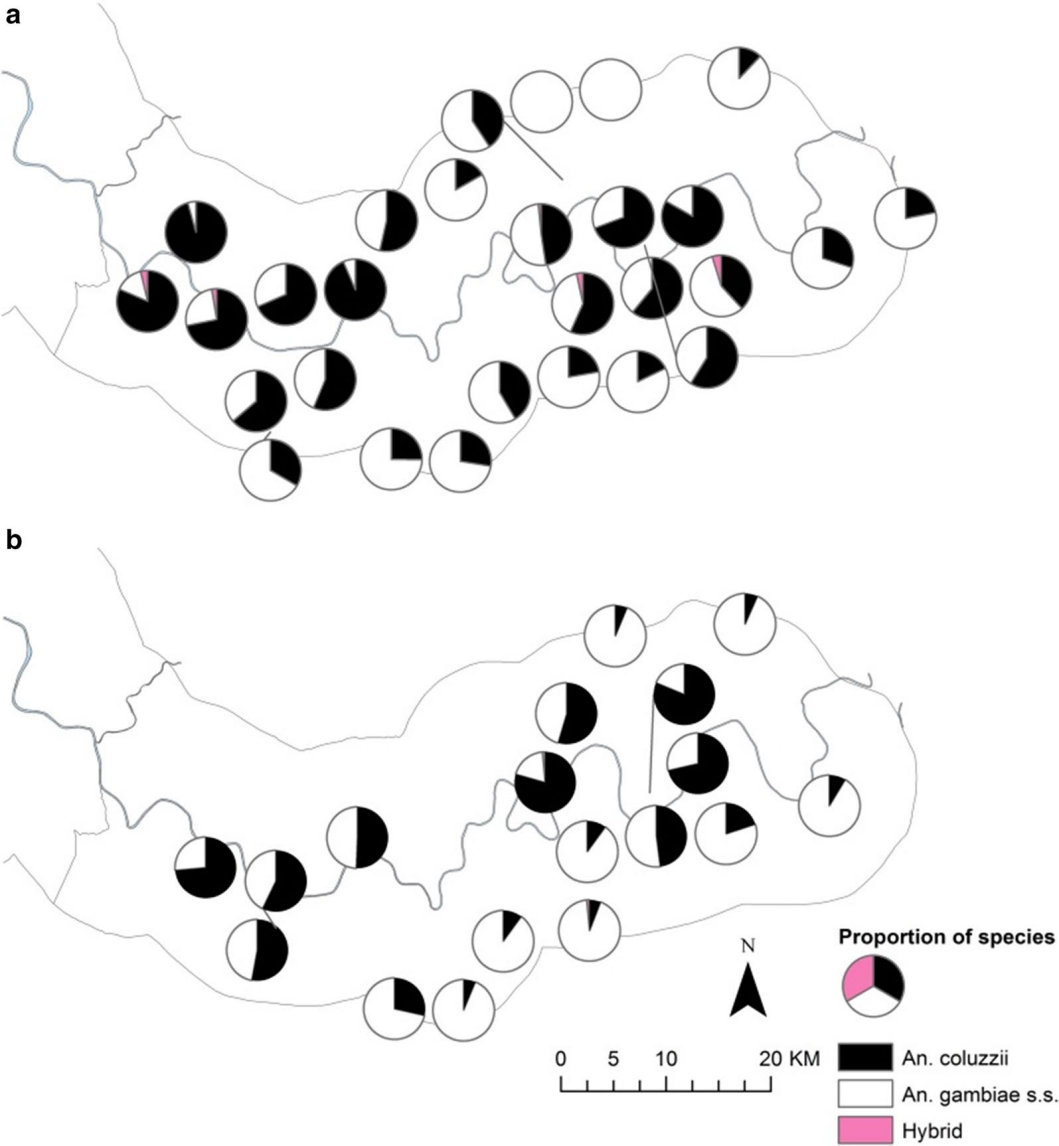
Distribution of members of the *An. gambiae s.l.* species complex (excluding *An. arabiensis*) in the study area during 2010 (**a**) and 2011 (**b**) transmission seasons. Pie charts show percentage *An. gambiae s.l.* species composition (excluding *An. arabiensis*) at CDC light trap sampling sites (excluding sampling sites with less than 10 mosquitoes caught in total across each transmission season)

**Fig. 5:**
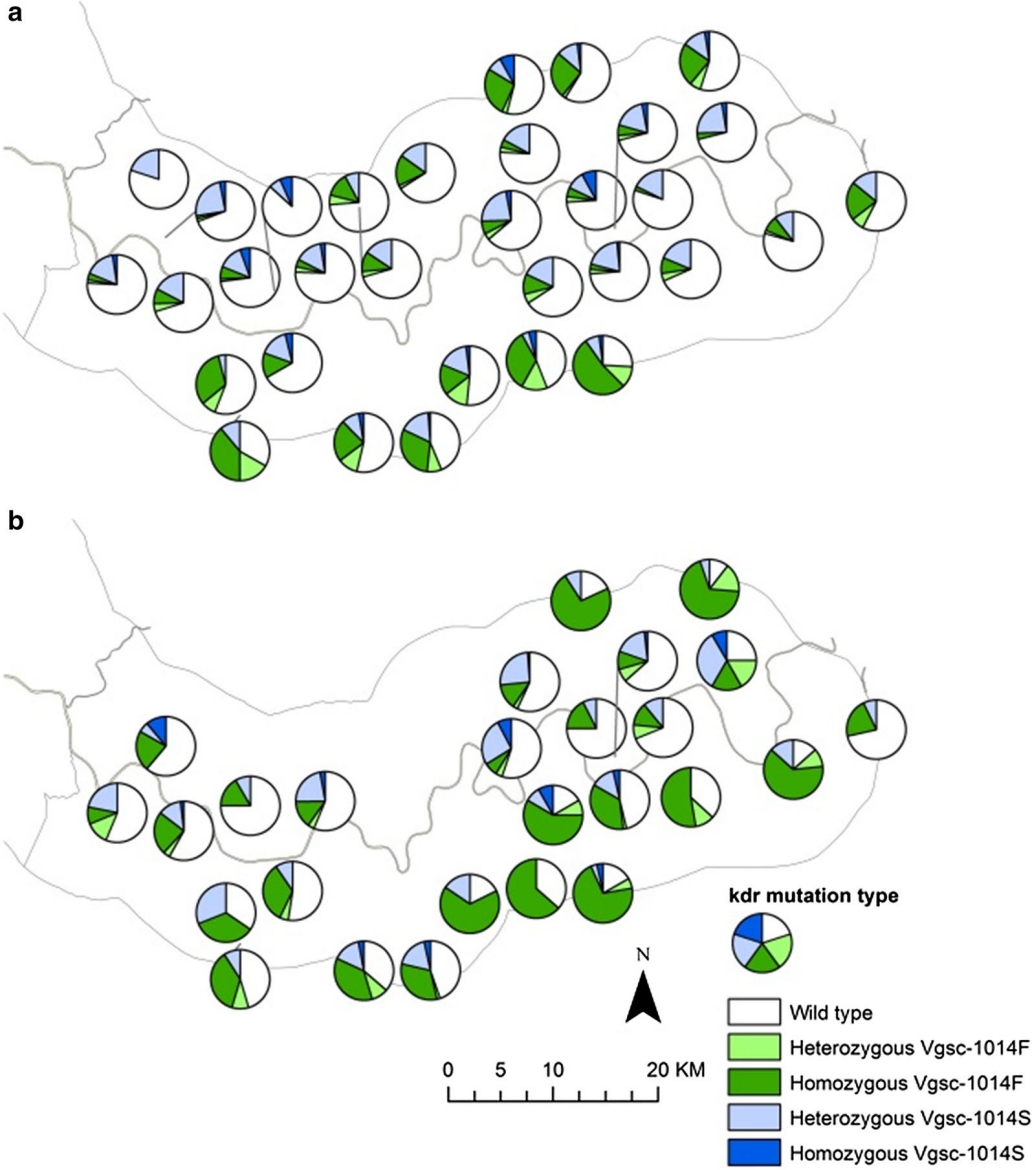
*Vgsc*-*1014* mutation status of *An. gambiae s.l.* in the study area during 2010 (**a**) and 2011 (**b**) transmission seasons. Pie charts show percentage wildtype, homozygous and heterozygous *Vgsc*-*1014F* and *Vgsc*-*1014S* mutations in *An. gambiae s.l.* complex at CDC light trap sampling sites (excluding sampling sites with less than 10 mosquitoes caught in total across each transmission season)
