# Supplementary material for: Emergence of knock-down resistance in the Anopheles gambiae complex in the Upper River Region, The Gambia, and its relationship with malaria infection in children
Source: Malar J. 2018 May 18;17:205. doi: 10.1186/s12936-018-2348-8 (PMC5960171; doi:10.1186/s12936-018-2348-8)
Supplement: Supplementary file 1 — Additional file 1. Characteristics of village clusters and proportion species composition during 2010 and 2011 transmission seasons. [file 12936_2018_2348_MOESM1_ESM.docx]

Additional file 1: Characteristics of village clusters and proportion species composition during 2010 and 2011 transmission seasons

| Village code | Sampling site | Study arm | Coordinates | | 2010 | | | | | 2011 | | | | |
| --- | --- | --- | --- | --- | --- | --- | --- | --- | --- | --- | --- | --- | --- | --- |
|  |  |  | Lat. | Long. | N | *An. arabiensis* | *An. gambiae* s.s. | *An. coluzzii* | Hybrid form | N | *An. arabiensis* | *An. gambiae* s.s. | *An. coluzzii* | Hybrid form |
|  | North bank | | | | | | | | | | | | | |
| 1 | Tuba Mandinka | IRS +LLIN | 13.43321 | -14.2502 | 103 | 75 (72.8%) | 13 (12.6%) | 15 (14.6%) | 0 | 4 | 3 (75.0%) | 0 | 1 (25.0%) | 0 |
| 2 | Mbye Kunda | LLIN | 13.37692 | -14.2806 | 373 | 316 (84.7%) | 3 (0.8%) | 44 (11.8%) | 0 | 277 | 194 (70.0%) | 41 (14.8%) | 42 (15.2%) | 0 |
| 3 | Changally Chewdo | IRS +LLIN | 13.36216 | -14.3253 | 19 | 13 (68.4%) | 0 | 3 (15.8%) | 0 | * |  |  |  |  |
| 4 | Kuraw Arafang | LLIN | 13.40808 | -14.421 | 128 | 103 (80.5%) | 1 (0.8%) | 22 (17.2%) | 0 | 20 | 12 (60.0%) | 4 (20.0%) | 4 (20.0%) | 0 |
| 5 | Jakaba | LLIN | 13.37311 | -14.3596 | 76 | 57 (75.0%) | 6 (7.9%) | 13 (17.1%) | 0 | 12 | 5 (41.7%) | 2 (16.6%) | 5 (41.7%) | 0 |
| 6 | Sare Jallow | IRS +LLIN | 13.45815 | -14.4268 | 10 | 10 (100%) | 0 | 0 | 0 | * |  |  |  |  |
| 7 | Tuba Wuli | IRS +LLIN | 13.43777 | -14.2397 | 38 | 29 (76.3%) | 6 (15.8%) | 3 (7.9%) | 0 | 3 | 3 (100.0%) | 0 | 0 | 0 |
| 8 | Jecka | LLIN | 13.53879 | -14.1112 | 37 | 27 (73.0%) | 9 (24.3%) | 0 | 0 | 5 | 3 (60.0%) | 2 (40.0%) | 0 | 0 |
| 9 | Medina Saho | LLIN | 13.47172 | -14.0974 | 291 | 264 (90.7%) | 16 (5.5%) | 11 (3.8%) | 0 | 81 | 59 (72.8%) | 10 (12.3%) | 12 (14.8%) | 0 |
| 10 | Limbanbulu Bambo | IRS +LLIN | 13.41971 | -14.1164 | 316 | 245 (77.5%) | 32 (10.1%) | 30 (9.5%) | 1 (0.3%) | 202 | 134 (66.3%) | 13 (6.4%) | 54 (26.7%) | 1 (0.5%) |
| 11 | Mureh Kunda | IRS +LLIN | 13.54888 | -14.0546 | 52 | 36 (69.2%) | 16 (30.8%) | 0 | 0 | 22 | 6 (27.3%) | 15 (68.2%) | 1 (4.5%) | 0 |
| 12 | Boro Dampha Kunda | IRS +LLIN | 13.41458 | -14.0251 | 276 | 227 (82.2%) | 14 (5.1%) | 20 (7.2%) | 0 | 138 | 90 (65.2%) | 9 (6.5%) | 39 (28.3%) | 0 |
| 13 | Musa Kunda / Kanapeh | IRS +LLIN | 13.55847 | -13.9412 | 120 | 82 (68.3%) | 29 (24.2%) | 4 (3.3%) | 0 | 20 | 5 (25.0%) | 14 (70.0%) | 1 (5.0%) | 0 |
| 14 | Foday Kunda | LLIN | 13.497 | -13.9263 | 117 | 110 (94.0%) | 5 (4.3%) | 2 (1.7%) | 0 | 13 | 9 (69.2%) | 2 (15.4%) | 2 (15.4%) | 0 |
| 15 | Tuba Buray / Kuvonkunding | LLIN | 13.463 | -14.1917 | 53 | 41 (77.4%) | 10 (18.9%) | 2 (3.8%) | 0 | 1 | 1 (100.0%) | 0 | 0 | 0 |
| 16 | Boro Modi Banni | LLIN | 13.43877 | -14.031 | 115 | 98 (85.2%) | 4 (3.5%) | 9 (7.8%) | 0 | 41 | 33 (80.5%) | 4 (9.8%) | 4 (9.8%) | 0 |
|  | South Bank | | | | | | | | | | | | | |
| 17 | Bolibana / Sare Batch | LLIN | 13.43679 | -13.7948 | 101 | 67 (66.3%) | 25 (24.8%) | 7 (6.9%) | 0 | 14 | 8 (57.1%) | 5 (35.7%) | 1 (7.1%) | 0 |
| 18 | Fantumbung | LLIN | 13.40275 | -13.8681 | 58 | 48 (82.8%) | 7 (12.1%) | 3 (5.2%) | 0 | 32 | 9 (28.1%) | 21 (65.6%) | 2 (6.3%) | 0 |
| 19 | Nema | LLIN | 13.43932 | -13.9828 | 223 | 202 (90.6%) | 3 (1.3%) | 15 (6.7%) | 0 | 124 | 75 (60.5%) | 14 (11.3%) | 35 (28.2%) | 0 |
| 20 | Kumbul | IRS +LLIN | 13.37917 | -13.9584 | 118 | 94 (79.7%) | 12 (10.2%) | 8 (6.8%) | 1 (0.8%) | 19 | 4 (21.1% | 12 (63.2%) | 3 (15.8%) | 0 |
| 21 | Perai | LLIN | 13.37738 | -14.0324 | 373 | 325 (87.1%) | 18 (4.8%) | 28 (7.5%) | 0 | 58 | 31 (53.4%) | 13 (22.4%) | 12 (20.7%) | 0 |
| 22 | Niji | IRS +LLIN | 13.29681 | -14.0781 | 156 | 48 (30.8%) | 88 (56.4%) | 19 (12.2%) | 1 (0.6%) | 91 | 23 (25.3%) | 63 (69.2%) | 4 (4.4%) | 1 (1.1%) |
| 23 | Koli Kunda | IRS +LLIN | 13.36431 | -14.0798 | 118 | 88 (74.6%) | 12 (10.2%) | 17 (14.4%) | 1 (0.8%) | 12 | 2 (16.7%) | 9 (75.0%) | 1 (8.3%) | 0 |
| 24 | Keneba | IRS +LLIN | 13.3009 | -14.1002 | 50 | 23 (46.0%) | 21 (42.0%) | 6 (12.2%) | 0 | 11 | 2 (18.2%) | 7 (63.6%) | 2 (18.2%) | 0 |
| 25 | Manpata Yel / Sare Musa | IRS +LLIN | 13.23037 | -14.221 | 185 | 101 (54.6%) | 62 (33.5%) | 21 (11.4%) | 0 | 33 | 19 (57.6%) | 10 (30.3%) | 4 (12.1%) | 0 |
| 26 | Sare Yero Cheke / Sare Sambo Lolo | LLIN | 13.24672 | -14.1883 | 91 | 51 (56.0%) | 29 (31.9%) | 11 (12.1%) | 0 | 59 | 43 (72.9%) | 15 (25.4%) | 1 (1.7%) | 0 |
| 27 | Tabajang | LLIN | 13.35163 | -14.4133 | 275 | 190 (69.1%) | 20 (7.3%) | 56 (20.4%) | 2 (0.7%) | 55 | 21 (38.2%) | 9 (16.4%) | 25 (45.5%) | 0 |
| 28 | Sare Sankuleh | IRS +LLIN | 13.29962 | -14.352 | 51 | 35 (68.6%) | 7 (13.7%) | 9 (17.6%) | 0 | 21 | 7 (33.3%) | 6 (28.6%) | 8 (38.1%) | 0 |
| 29 | Jalali Kunda / Sare Mansali | IRS +LLIN | 13.25416 | -14.3558 | 21 | 9 (42.9%) | 8 (38.1%) | 4 (19.0%) | 0 | 11 | 5 (45.5%) | 4 (36.4%) | 2 (18.2%) | 0 |
| 30 | Hella Kunda / Sare Momudu Afia | LLIN | 13.2809 | -14.3683 | 29 | 14 (48.3%) | 4 (13.8%) | 7 (24.1%) | 0 | 31 | 14 (45.2%) | 8 (25.8%) | 9 (29.0%) | 0 |
| 31 | Timbinto | LLIN | 13.36741 | -14.4639 | 166 | 137 (82.5%) | 4 (2.4%) | 22 (13.3%) | 1 (0.6%) | 33 | 25 (75.8%) | 3 (9.1%) | 5 (15.2%) | 0 |
| 32 | Taba Tafsir | IRS +LLIN | 13.28795 | -14.1535 | 103 | 62 (60.2%) | 24 (23.3%) | 17 (16.5%) | 0 | 45 | 15 (33.3%) | 27 (60.0%) | 3 (6.7%) | 0 |
| * No data from two villages (Changally Chewdo and Sare Jallow) in 2011 | | | | | | | | | | | | | | |
